# Urinary Deposites; Their Diagnosis, Pathology, Etc.

**Published:** 1852-01

**Authors:** 


### ARTICLE VII.

*Urinary Deposites: their Diagnosis, Pathology, and Therapeutical Indications.* By GOLDING BIRD, A. M., M. D., F. R. S., F. L. S. Second American, from the third revised and enlarged London edition. Philadelphia: Blanchard & Lea, 1851. (From the Publishers through S. C. Griggs & Co.)

This, the third edition, is amended and improved by additions which contain the most important discoveries in this department of science. The subject upon which this treats is of much importance to the practicing physician; but it has been, and is yet too much neglected. A proper knowledge of the subject gives much importance in regard to the pathology, prognosis, and treatment of certain diseases. Many obscure points in the pathology of disease, are rendered much more clear by the information given in this little work. The subject is one which should attract the attention of the medical profession, and we are happy that there are signs of the medical mind being turned in this direction, and hope that his little volume will be read and consulted by all who practice in the profession. J. McL.

